# Lateralized Changes in Language Associated Auditory and Somatosensory Cortices in Autism

**DOI:** 10.3389/fnsys.2022.787448

**Published:** 2022-03-01

**Authors:** Tara Deemyad

**Affiliations:** Department of Psychiatry, School of Medicine, University of Pittsburgh, Pittsburgh, PA, United States

**Keywords:** lateralization, autism, somatosensory cortex, auditory cortex, interneuron, circuit

## Abstract

Lateralized specialization of the two cerebral hemispheres is a fundamental structural hallmark of the human brain and underlies many cognitive functions and behavioral abilities. In typical developing individuals the influence of handedness on performance of various sensory modalities and the cortical processing has been well recognized. Increasing evidence suggests that several neurodevelopmental and psychiatric disorders such as bipolar disorder, schizophrenia, and autism spectrum disorders (ASD) are associated with abnormal patterns of cerebral lateralization. Individuals with ASD exhibit abnormal structural and functional lateralization of circuits subserving motor, auditory, somatosensory, visual face processing, and language-related functions. Furthermore, a high prevalence of atypical handedness has been reported in ASD individuals. While the hemispheric dominance is also related to functions other than handedness, there is a clear relationship between handedness and language-related cortical dominance. This minireview summarizes these recent findings on asymmetry in somatosensory and auditory cortical structures associated with language processing in ASD. I will also discuss the importance of cortical dominance and interhemispheric disruption of balance between excitatory and inhibitory synapses as pathophysiological mechanisms in ASD.

## Introduction

Despite the similarities in the macrostructure of the two brain hemispheres, each hemisphere has tendency to perform specific tasks. For example, neuropsychological and neuroimaging studies have revealed a strong bias toward left hemisphere representation of language and fine motor control of the hands (Kimura and Archibald, 1974; Springer et al., 1999), with a well-documented association between handedness and language lateralization (Annett, 1976). Right-sided brain damage is more likely to produce hemi-spatial attentional neglect, suggesting that visuospatial attentional abilities are represented in this hemisphere (Heilman and Van Den Abell, 1980). Although the mechanisms underlying functional lateralization are unknown, such lateralization in the brain structure and function is required for a wide range of higher order cognitive processes, including attention, motor control, language learning and tactile discrimination. Handedness (i.e., general preference for using the right or left hand) or dexterity (i.e., skill in performing tasks with the right or left hand) is one of the most recognizable functional asymmetries and is seen in both rodents and primates: there is a strong preference for using the right hand in humans (∼90%) (Hardyck and Petrinovich, 1977; Peters et al., 2006; Papadatou-Pastou et al., 2020) and the right forepaw in rodents (∼60%) (Waters and Denenberg, 1994; Guven et al., 2003). The hand dominance is well correlated with contralateral hemispheric specialization for fine motor behavior. Interestingly, the cumulative information from different studies suggests that the dominance in the functions of various other cortical areas is also associated with handedness. For instance, the possibility that the left-hemisphere controls language processing is about 1.5-times higher in right-handers compared to left-handers (Knecht et al., 2000a). Or the visual areas specialized for face recognition (i.e., fusiform face area) and perception of the human body (i.e., extrastriate body area and fusiform body area) are in the right hemisphere in right-handers (Willems et al., 2010). A recent meta-analysis has shown that genetic influences on handedness were associated with asymmetries in cortical thickness and surface areas, especially in language-related regions, which suggests a link between handedness and language during human development (Sha et al., 2021).

Abnormal patterns of brain lateralization have been linked to neurodevelopmental and psychiatric disorders such as autism spectrum disorders (ASD), schizophrenia and bipolar disorder. Autism is a neurodevelopmental disorder that is described by a broad range of cognitive and behavioral abnormalities. We have come a long way since Kanner (1943) and Asperger (1944) described autism for the first-time, as an abnormal social development as per Eugen Bleuler. Since then, the definition of autism has changed, and this disorder is now considered as a part of a spectrum of related conditions. Current diagnostic systems identify ASD based on two major criteria: social/communicative deficits and presence of repetitive behaviors. Also, a wide range of environmental, genetic, and biological factors have been implicated in the pathophysiology of autism (Mullins et al., 2016). Yet, regardless of its etiology, abnormal modulation of sensory inputs has been a pathognomonic feature of this disorder since its original description. About 95% of parents report some form of aberrant processing in at least one of the senses, such as hearing, vision and touch (Rogers and Ozonoff, 2005). Hyper- or hypo-reactivity to sensory input consider as a manifestation of restricted, repetitive patterns of behavior in these individuals (DSM-V) (Gnanavel and Robert, 2013). Difficulty and delay in developing language skills, perception of speech and non-verbal communication are also common in children with ASD. Developing comprehensive language and communication skills are contingent on intact sensory processing and integration, suggesting presence of aberrant information processing in one or multiple sensory modalities in ASD (Ayres and Mailloux, 1981; Mauer, 1999).

Proper functioning of cerebral cortex circuits is a fundamental requirement for higher order processing of information by the brain. As mentioned above, in typical developing individuals, the macroscopic organization of cortical hemispheres is lateralized for some functions or cognitive processes of the brain, such as language-related networks, visuospatial attention, and hand preferences (Gazzaniga, 1995; Gotts et al., 2013). In other words, each hemisphere of the brain seems to be specialized for processing of certain types of information. Interestingly, prevalence of left-handedness or ambidexterity dramatically increased by up to 3.5 times in ASD individuals compared to typical developing individuals (Markou et al., 2017). Furthermore, it has been suggested that atypical hemispheric specializations and poorer language skills in the autistic population might be associated with this particular pattern of handedness (Knaus et al., 2010, 2016; Finch et al., 2017). In particular, comparing gray matter volume of perisylvian language regions and language abilities in right- and left-handed typically developing and ASD individuals, showed that prevalence of the typical laterality is more evident in right-handed controls whereas, atypical lateralization of language is more prevalent among and left-handers with ASD (Knaus et al., 2010).

There is a large body of literature indicating the atypical cerebral asymmetries in ASD including frontal lobe organization (Sutton and Davidson, 2000; Sutton et al., 2005), parietal (Wang et al., 2017) and temporal lobes (Pereira et al., 2018). In this mini-review, the more recent studies on aberrant hemispheric lateralization in the somatosensory and auditory cortices and their relation to language processing in ASD individuals will be discussed. Furthermore, at the neuronal level, the normal performance of cortical circuits in mammals depends on preserving the balance between excitatory and inhibitory (GABAergic) synaptic activity (E/I balance). It has been hypothesized that autism can caused by E/I immbalance in neural circuits that mediate language and social behaviors (Casanova et al., 2003; Rubenstein and Merzenich, 2003; Uzunova et al., 2016). Here, some of the studies that have suggested E/I imbalance as an important underlying pathophysiology for cortical dysfunction in ASD and the possibility of asymmetric changes in inhibitory neurons in these cortical areas in ASD will be discussed. Finally, evidence that suggests a link between handedness and atypical cortical asymmetries observed in ASD will be presented.

## Lateralization in Language Associated Auditory Cortical Regions in Autism Spectrum Disorders

Left and right temporal lobes in the middle and superior temporal gyri are considered as primary auditory cortices. These areas are responsible for sensation of sound and lower level processing as well as higher order processing of speech. For example, lower level analysis of pitch is performed in the transverse temporal gyrus (Warrier et al., 2009) and higher order language processing, comprehension, and the semantic content occurs in the angular gyrus and inferior parietal lobe (Xu et al., 2005; Hartwigsen et al., 2015).

Existence of the brain lateralization in audition and cortical auditory areas has been well established. Neuropsychological studies as well as functional and structural brain imaging methods have shown that left auditory cortex has the main role in speech processing and the right side plays a more dominant role in music processing (domain-specific lateralization) (Zatorre et al., 2002). Other studies suggest that the left auditory cortex is more dedicated to processing rapid changes in pitch while the right side processes small changes in pitch (parameter-specific lateralization). A recent study showed that in addition to the cortex, subcortical structures might also contribute to the hemispheric dominance for speech processing (Guadalupe et al., 2021).

Impairment of auditory and speech processing are among the hallmark features of autism. In particular, ASD individuals show decreased sensitivity to human voice without impairment in responding to other non-vocal stimuli (Klin, 1991, 1992; Dawson et al., 2004; Kuhl et al., 2005). Asymmetric structural change and atypical processing of vocal pitch at both lower and higher auditory processing centers may contribute to poor speech perception in ASD individuals. A recent Functional MRI study has shown an exclusive atrophy in the left temporal lobe in high functioning autistic individuals compared to normal control individuals (Pereira et al., 2018). An earlier study showed a reduction in the activity of left posterior insular cortex in ASD individuals during the auditory phrase-recognition task; an area that receives inputs from different parts of the auditory cortex (primary and association cortices) (Anderson et al., 2010). Finally, a recent study found that sustained neuronal field activity in the left transverse temporal gyrus (triggered by periodic sounds and recorded by magnetoencephalography), was delayed and displaced in pitch processing (Stroganova et al., 2020).

Although several studies have indicated a systematic correlation between handedness and hemispheric language dominance, none of the above studies have considered the effect of handedness in their observed lateralization. For instance, using functional transcranial Doppler sonography, it has been shown that language dominance (using a word-generation task) is linearly correlated with the degree of handedness in healthy individuals (Knecht et al., 2000b; Basic et al., 2004). Other studies have shown that left-handed neurotypical individuals would have weaker lateralization compared to the right-handed group in language tasks (Johnstone et al., 2020; Bruckert et al., 2021; Woodhead et al., 2021). Future studies are required to address the question of whether impairment in human voice processing in ASD individuals is associated with the dominant hemisphere or is simply related to dysfunction in the left hemisphere.

Another important open question to be addressed in ASD individuals is the developmental trajectory of the impairment in cortical thickness and structure, interhemispheric connectivity, vocal processing, and the time of deviation from the neuronal auditory/speech pathway in typical developing children. Interhemispheric connectivity between right and left auditory cortices has been shown to play a role in speech perception and phonetic categorization in typical developing adults. While interhemispheric connections are reduced in ASD individuals (Anderson et al., 2011; Zhu et al., 2014; Lee et al., 2016), its developmental trajectory in ASD subjects and its effect on early sensory processing and speech perception remains to be explored.

## Lateralization in Language Associated Somatosensory Cortical Regions in Autism Spectrum Disorders

Cerebral somatosensory areas are located in the postcentral gyrus. These parts of the brain receive and process the information from everywhere in the body regarding touch, pain, and vibration. The primary somatosensory area receives stimuli from contralateral half of the body, whereas the secondary somatosensory area receives the bilateral inputs from the entire body. Additionally, because of the callosal connectivity of the trunk map, areas 3b and 1 of primary somatosensory cortex in primates and humans receive bilateral inputs from the body midline (Fabri et al., 2005).

Autism spectrum disorders individuals frequently show various forms of somatosensory abnormalities. The most recognized abnormalities are hyperesthesia or hypoesthesia to light touch, temperature and pain (Courchesne et al., 2005). Tactile over-reactivity is also significantly correlated with social impairments and communication in children with ASD (Orefice, 2020). Importantly, somatosensory feedback from the facial skin and muscles involved in producing sound/speech (i.e., in pharynx and larynx) plays a fundamental role in speech articulation (Tremblay et al., 2003; Bouchard et al., 2013; Schomers and Pulvermuller, 2016) and perception (Mottonen et al., 2005; Skipper et al., 2007; Ito et al., 2009; Nasir and Ostry, 2009; Correia et al., 2015; Bartoli et al., 2016). Indeed, using this feedback gives some deaf individuals the ability to generate comprehensible speech (Tremblay et al., 2003; Fletcher, 2021). Remarkably, the efficiency of motor control of the speech articulators relies on accurate anticipation of the somatosensory reafference (Bartoli et al., 2016). Future studies are required to investigate whether a dysfunction in the somatosensory cortex of ASD individuals and abnormal somatosensory feedback plays a role in language production and perception deficits observed in ASD.

Nevertheless, our knowledge on tactile lateralization in ASD is limited compared to visual and auditory modalities. Using short-latency evoked potentials, it has been shown that in ASD individuals stimulation of the left median nerve results in an over-reactive response in the right primary somatosensory cortex compared to the response of the left primary somatosensory cortex to right median nerve stimulation (Miyazaki et al., 2007). More recently, an asymmetric increase in the gray matter volume in the left postcentral gyrus (i.e., the primary somatosensory area) in ASD individuals has been shown, using an optimized method of voxel-based morphometry analysis on structural magnetic resonance imaging data (Wang et al., 2017). Surprisingly, voxel-based morphometry analysis of functional and structural MRI results from 22 adolescents and young adults with ASD and 29 normal controls showed an aberrant decrease in gray matter thickness of postcentral gyrus in the left hemisphere of ASD individuals (Pereira et al., 2018). While the discrepancy between these two studies could be due to the heterogeneity of samples among the subgroups of ASD subjects and/or within the spectrum and differences in calculation methodologies (Pereira et al., 2018), one cannot rule out the role of hemispheric dominance in these differences since this variable was not considered in these studies. When it comes to involvement of somatosensory cortices in speech function, considering the hemispheric dominance and handedness becomes even more important. For instance, a neuroimaging study has shown that in neurotypical individuals, viewing another person’s articulatory actions preferentially activates the dominant primary somatosensory cortex (distinguished only based on handedness) in a somatotopic manner (Mottonen et al., 2005). Verbal and non-verbal lateralization is present in haptics stimuli. It has been shown that the non-dominant left hand is significantly better in discriminating nonsense shapes, whereas dominant right hand is superior in discrimination of letters (Stoycheva et al., 2020). Therefore, there is a need to better understand lateralization in cortical circuits and their input/outputs and its relation to handedness and/or dominant and non-dominant cortices.

## Lateralization in Cerebral Networks

**FIGURE 1:**
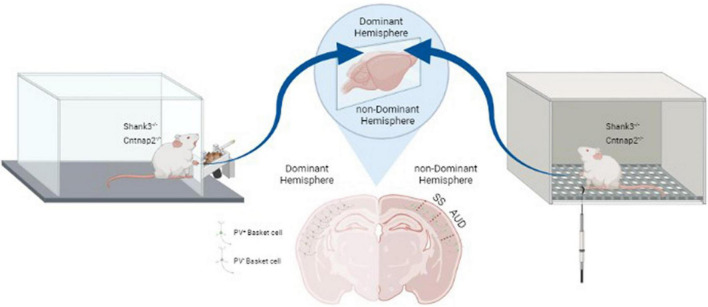
Relation between lateralized changes in PV expression and behavioral responses in Shank3^–/–^ and Cntnap2^–/–^ mouse models of ASD. Handedness was determined based on paw-preference for a food retrieval paradigm (left panel). PV expression was decreased in interneurons of the dominant somatosensory and auditory cortices (middle panel). Correlated with this decrease in inhibitory inputs, sensitivity of the dominant paw to mechanical stimuli was increased as measured by von Frey hair test (right panel) (Adapted from Deemyad et al., 2021). SS: Somatosensory area; AUD: Auditory area.

Although significant progress has been made in unraveling the structural and functional lateralization deficit in ASD, we know little about differences between the two hemispheres in terms of neuronal structures and circuits. Normally, excitatory Glutamatergic and inhibitory GABAergic neurons in cortical networks are wired in a manner to properly balance excitatory and inhibitory synaptic inputs to cortical neurons. Interneurons and in particular the ones expressing parvalbumin (PV), play an important role in maintaining this balance, which is critical for proper circuit activity, efficient coding of information and higher brain functions such as cognitive flexibility, attention, and social interaction (Mederos and Perea, 2019). Recent studies in several ASD mouse models have shown a decrease in PV cell numbers in different cortical areas and similar findings have been shown in postmortem brain of individuals with ASD. Furthermore, it has been shown that this decrease is in fact due to a decrease in the expression of PV in these interneurons rather than a change in the actual number of cells. Interestingly, two studies investigated interhemispheric differences of this change in PV cells. The first study (Gogolla et al., 2009) showed a lateralized reduction in the number of PV cells in the cerebral cortex of two ASD mouse models: adult mice with neuroligin-3 mutation (NL3^–/–^) or those exposed to Valproic Acid (VPA). An interesting feature of this study was that although ASD was generated with different mechanisms (i.e., presynaptic mechanism in NL3^–/–^ and postsynaptic in VPA model), they showed similar asymmetric changes in alteration in inhibitory interneuron networks. However, the changes in interneurons seem to be more complicated as more recent studies have shown that rather than a loss in PV cells, there is a decrease in the expression of PV by these interneurons (refs). In a recent study with double labeling by anti-PV antibody and a biotinylated lectin (to label basket cells) a robust decrease in PV expression was shown in the somatosensory cortex of two mouse models of ASD [i.e., mutations in contactin-associated protein-like 2 (Cntnap2^–/–^) or SH3 and multiple ankyrin repeat domains 3 (Shank3^–/–^)] (Deemyad et al., 2021). Furthermore, it was shown that this change was limited to one hemisphere, either right (36% of mice) or left (63% of mice) and was strongly correlated with the dominant hemisphere somatosensory cortical area based on handedness of these mice (Deemyad et al., 2021). To test their handedness, animals had to grab food pellets from a slit by their forepaws and depending on which forepaw was used the majority of the time, they were divided into right or left handed. Finally, the observed changes in PV cells in the dominant hemisphere was strongly correlated with hypersensitivity to mechanical tactile stimuli in the dominant paw (see Figure 1). It is currently unclear as to how the observed lateralization in PV expression in interneurons affects the inhibition, neuronal circuits and whether it is associated with alteration in E/I balance in local circuits. However, since PV cells innervate up to 200 pyramidal cells in the cortex, even a slight shift in the number of PV^+^ interneurons could potentially have a dramatic impact on cortical microcircuits and their function. Future studies are also required to clarify at which point during the early developmental period the lateralized changes in PV cells occur in ASD models and how it progresses over time.

As mentioned in the previous section, a systematic correlation has been shown between handedness and hemispheric language dominance. Interestingly, a similar lateralized decrease in PV expression has also been reported in the auditory cortex of Shank3^–/–^ and Cntnap2^–/–^ mice (Deemyad et al., 2021). Verbal communication is critically depending on precise processing of auditory information and perceiving the meaning of sounds. Such precise processing of auditory information shows hemispheric deficits in ASD (Marco et al., 2011; Tietze et al., 2019). For instance, an fMRI study on individuals with Asperger’s syndrome has shown weaker activation in the dominant auditory hemisphere when compared with typically developing controls (Tietze et al., 2019). It was proposed that such changes in auditory processing in the dominant hemisphere underlie the speech perception problems in Asperger’s syndrome. Possible lateralized changes in PV expression in the dominant auditory cortex and its role in language development remain to be studied in ASD individuals.

## Conclusion

In conclusion, it seems reasonable to consider lateralized cortical changes observed in ASD mouse models and ASD individuals in future studies, taking into account dominance of the hemispheres. For example, future studies on contribution of these two sensory modalities to language production and perception should consider methods such as handedness of subjects to identify the dominance of the language associated cortical areas. Future Studies are also required to address the effect of lateralized decrease in PV expression in interneurons on neuronal circuits and E/I balance in different cortical areas. If specific patterns of change are identified in cortical circuits, they could be used for early detection of affected cortical areas in ASD subjects and provide the means for possible prediction of correlated behavioral patterns. Finally, the developmental trajectory of the observed lateralized cerebral aberrations and its role in developing ASD behaviors has not been studied. Such studies could identify the critical period for instigating effective interventions.

## Author Contributions

The author confirms being the sole contributor of this work and has approved it for publication.

## Conflict of Interest

The author declares that the research was conducted in the absence of any commercial or financial relationships that could be construed as a potential conflict of interest.

## Publisher’s Note

All claims expressed in this article are solely those of the authors and do not necessarily represent those of their affiliated organizations, or those of the publisher, the editors and the reviewers. Any product that may be evaluated in this article, or claim that may be made by its manufacturer, is not guaranteed or endorsed by the publisher.
